# Genome-wide identification and expression profiling of long non-coding RNAs in auditory and vestibular systems

**DOI:** 10.1038/s41598-017-08320-3

**Published:** 2017-08-17

**Authors:** Kathy Ushakov, Tal Koffler-Brill, Aviv Rom, Kobi Perl, Igor Ulitsky, Karen B. Avraham

**Affiliations:** 1 Department of Human Molecular Genetics and Biochemistry, Sackler Faculty of Medicine and Sagol School of Neuroscience, Tel Aviv University, Tel Aviv, 6997801 Israel; 2 Department of Biological Regulation, Weizmann Institute of Science, Rehovot, 7610001 Israel; 3 Blavatnik School of Computer Science, Tel Aviv University, Tel Aviv, 6997801 Israel

## Abstract

Mammalian genomes encode multiple layers of regulation, including a class of RNA molecules known as long non-coding RNAs (lncRNAs). These are >200 nucleotides in length and similar to mRNAs, they are capped, polyadenylated, and spliced. In contrast to mRNAs, lncRNAs are less abundant and have higher tissue specificity, and have been linked to development, epigenetic processes, and disease. However, little is known about lncRNA function in the auditory and vestibular systems, or how they play a role in deafness and vestibular dysfunction. To help address this need, we performed a whole-genome identification of lncRNAs using RNA-seq at two developmental stages of the mouse inner ear sensory epithelium of the cochlea and vestibule. We identified 3,239 lncRNA genes, most of which were intergenic (lincRNAs) and 721 are novel. We examined temporal and tissue specificity by analyzing the developmental profiles on embryonic day 16.5 and at birth. The spatial and temporal patterns of three lncRNAs, two of which are in proximity to genes associated with hearing and deafness, were explored further. Our findings indicate that lncRNAs are prevalent in the sensory epithelium of the mouse inner ear and are likely to play key roles in regulating critical pathways for hearing and balance.

## Introduction

Advances in next-generation sequencing (NGS) in the past decade have boosted our understanding of the genome and have revealed a plethora of previously unknown non-coding RNAs. One such class of recently discovered RNA molecules is the long non-coding RNAs (lncRNAs), which, at greater than 200 nucleotides (nt) in length, have been categorized based on their size. Although they are spliced, polyadenylated, and capped, similar to messenger RNAs (mRNAs), lncRNAs have no recognizable coding potential and are expressed in a cell-specific manner; a minority of them is also highly conserved^1^. The ability of lncRNAs to interact with different molecules, including DNA, RNA, and proteins, allows them to impose an additional regulatory layer on the cell’s genetic program. Since their discovery, lncRNAs have been extensively studied and associated with a growing number of organ systems, such as the heart^2^ and eye^3^, as well as implicated in diseases, such as cancer^4^ and most recently, celiac disease^5^.

One key area that has not been well studied with respect to lncRNAs is the inner ear, responsible for both hearing and balance, and when involved in disease, often leads to deafness and imbalance. The sensory systems responsible for hearing and balance are the auditory and vestibular systems, respectively. The genetic mechanisms operating in these systems rely critically on complex genetic programs. Although these systems have extensive similarities, there are structural and functional differences as well. In the auditory system, the organ of Corti in the cochlea contains the sensory epithelium. The vestibular system contains five organs, including three semicircular canals with a cristae sensory epithelium for detecting angular acceleration by fluid motion, and the saccule and the utricle, which contain the macula sensory epithelium for detecting linear acceleration owing to gravity. The development of the inner ear is a dynamic biological process, eventually leading to the formation of complex tissue enabling hearing and balance, which has been well characterized in the mouse^6, 7^. In the cochlea, the majority of progenitor cells of the sensory epithelium exit the cell cycle by embryonic day 14 (E14) and subsequently, a differentiation gradient leads to formation of the cells of the organ of Corti^7, 8^. The sensory and non-sensory structures continue to mature after birth, with the onset of hearing initiated at post-natal day (P)12, and functional by P15^9^.

Since we predict that similar to other organ systems, there is a need for a specialized regulatory layer that includes lncRNAs, we set out to comprehensively explore lncRNAs in the inner ear. To this end, we defined a set of lncRNAs in the sensory epithelium of the mouse cochlea and vestibule by RNA-sequencing (RNA-seq) and evaluated their expression at two stages prior to the onset of hearing, E16.5 and P0. Our results indicate that over 3000 lncRNAs are expressed at these stages in the inner ear, with specific expression patterns during development in the hair and supporting cells and in the stria vascularis. Moreover, we have identified a subset of novel lncRNAs, strengthening the hypothesis of a crucial role for these genomic transcripts as regulators of the auditory and vestibular systems.

## Results

### RNA-seq of sensory epithelium reveals the inner ear transcriptome

**Figure 1:**
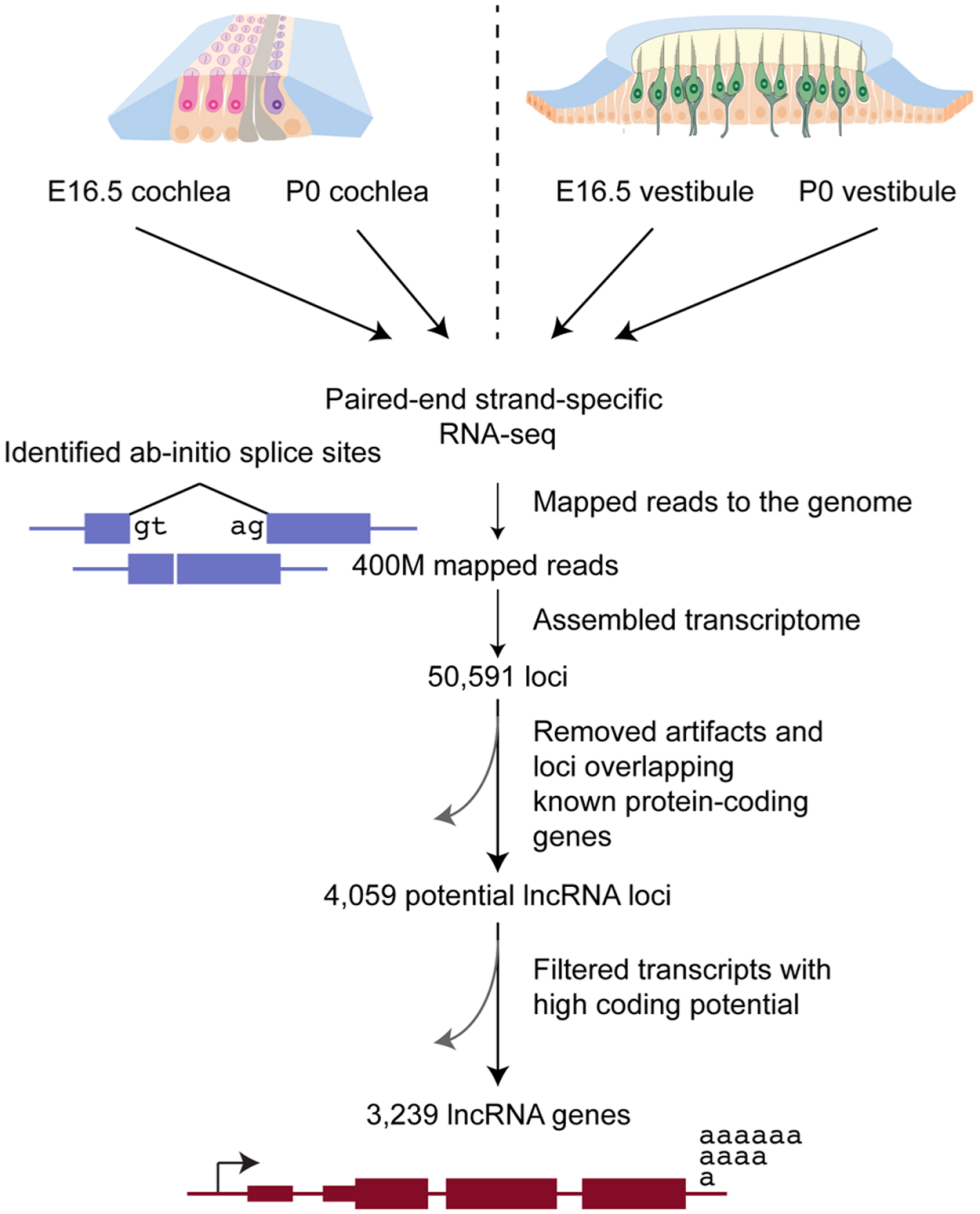
The long noncoding transcriptome of the inner ear sensory epithelium. The experimental and bioinformatic pipeline used for identifying lncRNAs in the inner ear. Extended details regarding bioinformatic programs used are found in the Methods section. Cochlea image modified from ref. 64.

In order to identify lncRNAs in the auditory and vestibular sensory epithelium, we sequenced polyadenylated RNA molecules at two developmental stages, E16.5 and P0. For each age, we isolated RNA from sensory epithelium derived from 20 inner ears of 10 wild-type C57BL/6 mice. For the cochlea, the sensory epithelium consisted of the organ of Corti, including hair cells, supporting cells, and cells of the greater and lesser epithelial ridges. For the vestibule, the sensory epithelium was derived from five sensory patches: saccular macula and utricular macula and the anterior, posterior, and lateral cristae and included hair and supporting cells. The collected tissue from both the cochlea and vestibule included mesenchymal and neuronal cells. cDNA libraries were generated and subjected to paired-end strand-specific RNA-seq (Fig. 1).

**Figure 2:**
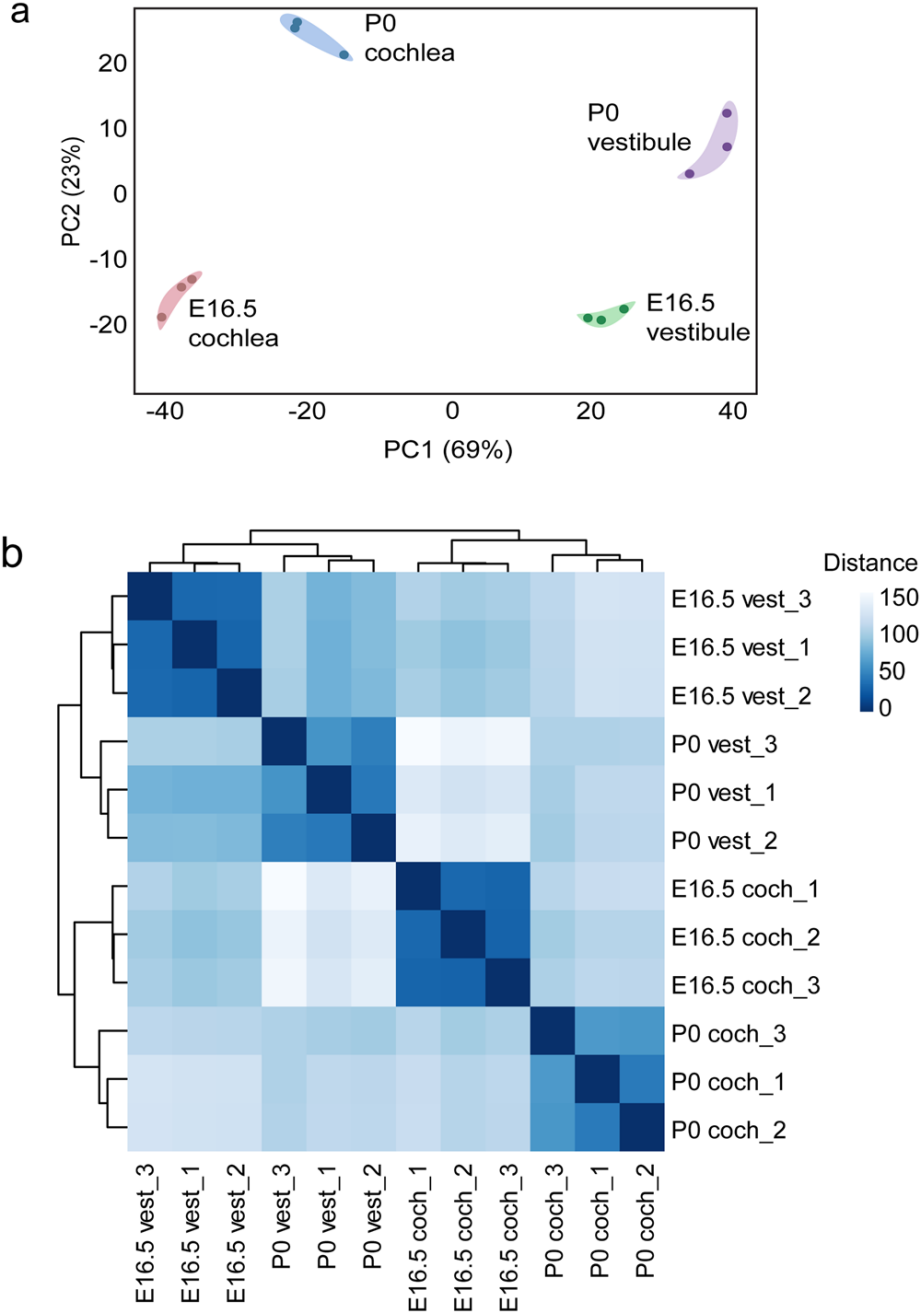
High-resolution transcriptome analysis of mouse inner ear sensory organs. (**a**) Principal component analysis (PCA). PC1 (X-axis), which accounts for 69% variance, divides the vestibule (green and purple) from the cochlea (blue and red), whereas PC2 (Y-axis), which represents 23% of the variance between the samples, separates tissue from E16.5 and P0. (**b**) Hierarchical clustering analyses with heatmap performed on the twelve inner ear samples. The color code refers to the Euclidian distance used for clustering (the maximum similarity is denoted by dark blue).

To provide an overall estimation of the transcriptome changes occurring during the maturation of mouse auditory sensory organs, we compared the global expression across all four conditions. As expected, the complete transcriptional profiles, both for coding and non-coding genes, of the four conditions were distinct, with replicates clustering together (Fig. 2a) and samples from a similar stage or tissue type being similar to each other (Fig. 2b). The majority of the variability, represented by the first principal component, was between distinct tissues; the second principle component separated between the developmental advancement of the auditory and vestibular organs. Overall, we developed a robust protocol, which enables explicit estimation of auditory and vestibular transcriptomes.

### Novel lncRNAs are identified among auditory and vestibular transcripts

The RNA-seq data were analyzed by the computational pipeline we developed^10^ for discovering lncRNAs (Fig. 1; see Methods for details). Briefly, reads were aligned to the mouse genome (mm10 assembly) using STAR based on splice junctions from the Ensembl database^11^. The transcriptome was assembled using CuffLinks^12^ and contained 179,373 transcripts from 50,591 genes. It was further processed using the pipeline for lncRNA annotation from RNA-seq data (PLAR) method^10^. We identified putative lncRNAs by considering the signatures of nucleotide changes across species, homology with known proteins and protein domains, as well as potential ORFs. We note that it is possible that some of these transcripts are translated to produce short and poorly conserved ORFs, but its unlikely that many accumulate to substantial levels^13^. Finally, transcript abundance was estimated using the RSEM method^14^.

**Figure 3:**
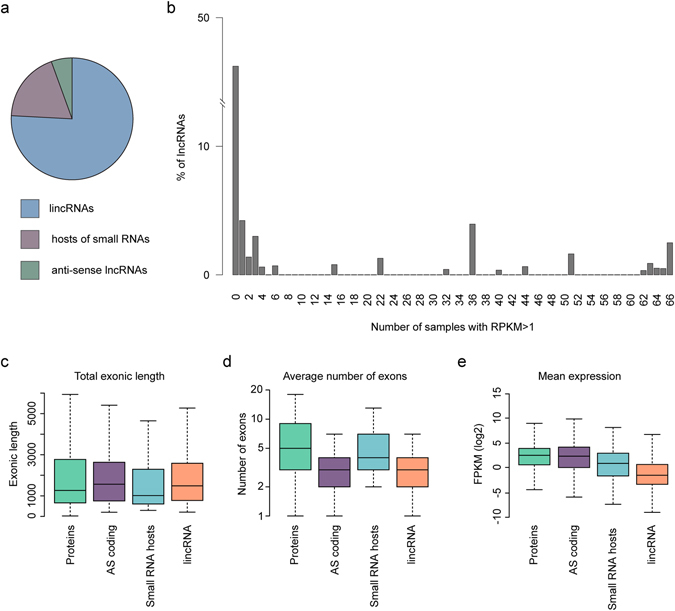
Properties of inner ear transcripts. (**a**) Global overview of the transcript type in the developing inner ear. The pie chart displays lncRNAs transcripts. (**b**) Expression levels of the lncRNAs identified in the inner ear sensory epithelium were evaluated in 66 RNA-seq samples profiled in ENCODE and representing various mouse embryonic and adult tissues. For each lncRNA gene, the number of samples in which it was expressed at FPKM ≥ 1 is shown (e.g., the lncRNAs in column #66 are ubiquitously expressed in ENCODE data). Box plots of (**c**) exon size distribution, (**d**) distribution of the average number of exons, and (**e**) gene expression represented in FPKM for protein-coding, antisense (AS) coding, small RNA hosts, and lincRNA transcripts.

After filtering, we identified 6,318 lncRNA transcripts from 3,239 distinct genes. A total of 4,792 of the transcripts were intergenic (long intervening noncoding RNAs, or lincRNAs), 350 hosts of small RNAs, and 1,176 lncRNAs antisense to protein-coding genes (antisense transcripts, Fig. 1, Fig. 3a, Supplementary Table S1). We note that the PLAR pipeline does not permit lncRNAs that overlap introns of protein-coding genes on the same strand (due to the difficulty in their annotation). Of the lncRNA transcripts, 1,460 did not overlap those annotated in the GENECODE release 24/Ensembl release 84. When grouping overlapping transcripts, 721 distinct lncRNA genes did not overlap GENCODE annotations and were considered novel (Supplementary Table S2). 46% of the lncRNAs we identified in the inner ear were not expressed at appreciable levels (RPKM > 1) in any of 66 samples of various embryonic and adult tissues profiled by ENCODE, and many others were expressed only in a handful of tissues (Fig. 3b; Supplementary Table S1).

The lincRNA and the antisense transcripts have a similar length to protein-coding genes (Fig. 3c). The average number of exons in lincRNAs and in antisense transcripts is similar; however, lincRNAs have significantly fewer exons than protein coding transcripts and hosts of small RNAs. LincRNAs and antisense transcripts typically have 2-3 exons, whereas protein-coding and small RNA hosts have, on average, 4–5 exons (Fig. 3d). In terms of expression, based on fragments per kilobase per million of reads (FPKM), lncRNAs were consistently expressed at an order of magnitude lower levels than mRNAs, with a median of 0.3 FPKM compared with 5.9 FPKM of coding transcripts (Fig. 3e).

A recent transcriptomics study of human inner ear tissue led to the identification of non-coding RNAs^15^. To assess the level of conservation of lncRNA expression, we searched for orthologous genes between our lncRNA set and lncRNAs annotated in human, where orthology was determined both by sequence similarity and by synteny (Supplementary Methods). The human database that we searched contained 7,109 lncRNAs expressed in the inner ear. Out of the 3,239 lncRNAs identified in our data, 139 had sequence similarity to a human lncRNA, 1,049 were syntenic to a lncRNA, and 101 had both qualities. That is, the majority of mouse auditory lncRNA loci lack recognizable human homologs. After resolving multiple matching, 93 orthologous pairs remained. These were further clustered according to their expression pattern across species, tissues and ages, into three groups of 22, 28, and 43 lncRNAs (Supplementary Fig. S1, Table S3). The first group contained lncRNAs highly expressed in human, the majority of which were highly expressed in mouse as well. The second group contained lncRNAs highly expressed in the mouse ear, with low to intermediate expression in the human ear. The third group contained lncRNAs with low to intermediate expression in both species. Taken together, the properties of the inner ear lncRNAs, in terms of gene architecture, expression levels, tissue specificity and conservation are similar to the properties of lncRNAs discovered in other tissues.

### Differential expression of genes is greater in the cochlear sensory epithelia

With the goal of identifying and functionally validating novel genetic elements underlying inner ear development, we evaluated the global expression changes at two stages, E16.5 and P0. To determine the differential expression, we performed a pairwise differential expression analysis between two tissues and two developmental stages using the DESeq. 2 package^16^.

At both developmental stages of the mouse inner ear, the most abundantly expressed lncRNAs are the imprinted H19 gene and 1700012D14Rik (Supplementary Table S1). Other known and partially characterized genes, such as Fendrr, Xist, and Malat1, are also expressed at considerable levels.

**Figure 4:**
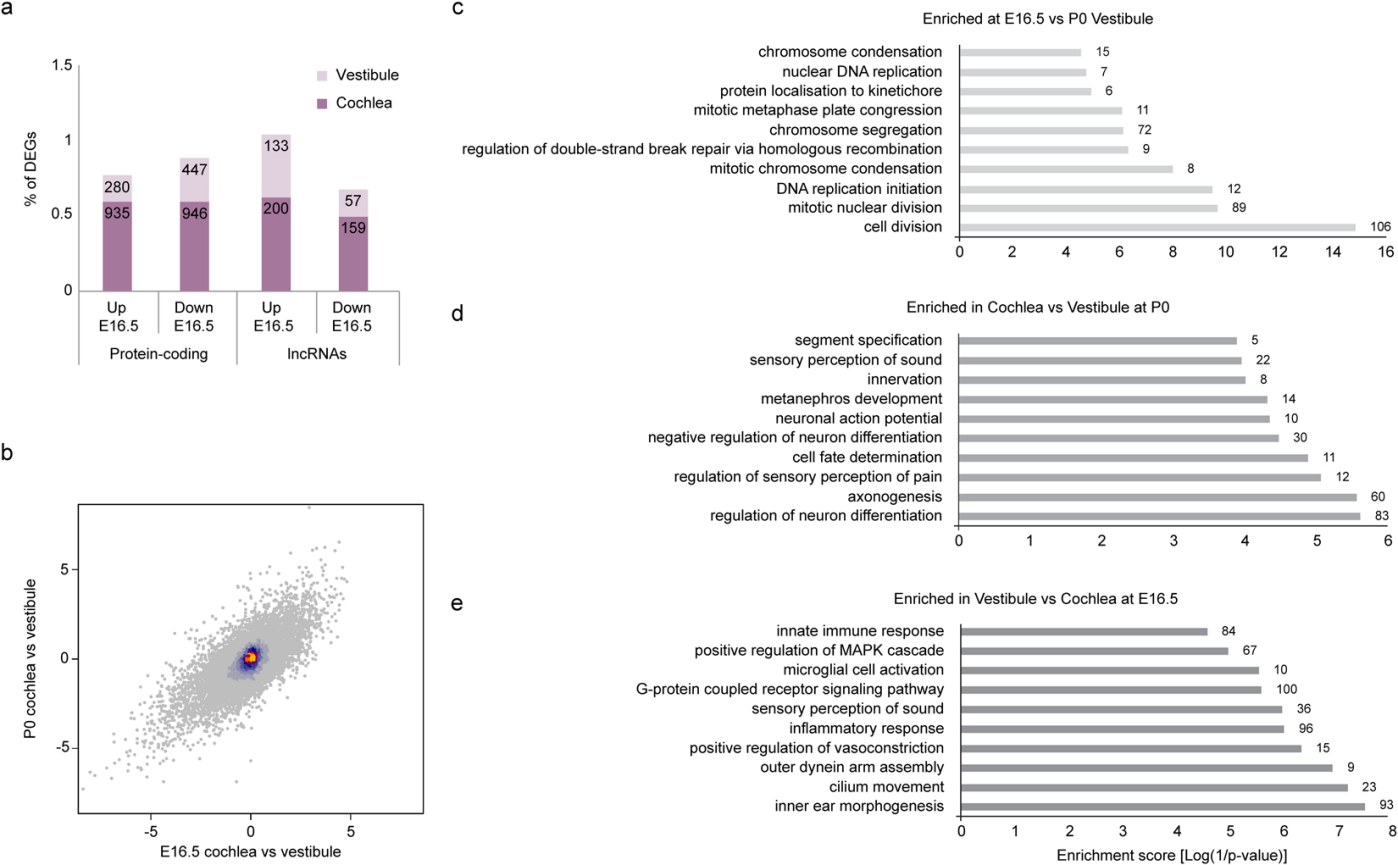
Functional annotation of differentially expressed genes (DEGs). (**a**) Bar graph comparing the number of DEGs (adjusting P < 0.05, a fold change of at least 2) at E16.5, in the vestibule compared to cochlea. The percentage was calculated from the total coding and non-coding transcripts, 15,916 and 3,239, respectively. (**b**) Scatter plot of pairwise log2 fold changes for the cochlea vs vestibule DEGs between E16.5 (x-axis) and P0 (y-axis). (**c**–**e**) Top ten TopGO functional gene ontology (GO) annotations for the DEGs. Bar graphs show all three GO information categories (biological processes, molecular functions, and cellular components). The abscissa represents the number of DEGs. All GO categories listed exhibit enrichment with p < 0.05. (**c**) Enriched in vestibule at E16.5 vs P0. (**d**) Enriched at E16.5 in vestibule vs cochlea. (**e**) Enriched at P0 in cochlea vs vestibule.

When comparing transcripts that are differentially expressed (adjusted P < 0.05, a fold change of at least 2) between the tissues and throughout the development of the inner ear, the percentage of differential protein-coding and lncRNA genes is similar (Fig. 4a,b). Strikingly, the number of differentially expressed genes (DEGs) was significantly higher in the cochlea compared to the vestibule. This might indicate that there are differences in the complexity of the tissues or it might validate the development-specific timing of events. The differentially expressed mRNAs and lncRNAs are listed in Supplementary Tables S4 and S5, respectively. Fcrlb (fold change: 80.08) and XLOC_008456 (fold change: 17.43) are mRNA and lncRNA genes, respectively, which are the most enriched genes in the cochlea at E16.5. In summary, major differences exist in the number and the nature of the DEGs, both within the tissue and at the developmental stage.

### Functional categories of differentially expressed genes

To further reveal the major biological processes that are activated in the vestibule and the cochlea throughout development, we used gene ontology (GO) enrichment analysis^17^. GO categories corresponding to various aspects of the cell cycle and DNA metabolic processes (e.g. “mitotic metaphase plate congression”, “chromosome segregation” and “DNA replication initiation”) were enriched in genes with increased expression at E16.5 as compared with P0 in both tissues (Fig. 4c, Supplementary Table S6).

Interestingly, when comparing the GO terms between the cochlea and vestibule, we noted several trends. Throughout cochlear development, there was elevated expression of genes associated with neuronal processes (e.g. GO terms “axonogenesis”, “neuronal action potential” and “regulation of membrane potential”, Fig. 4d). At E16.5 more regulatory processes are involved in ion transmembrane transport, whereas after birth, the enriched categories shifted to “negative regulation of neuron differentiation” and “sensory perception of sound”. Nonetheless, throughout vestibular development, there is enrichment for annotations related to the immune system (e.g. “inflammatory response” and “innate immune response”), as well as “cilia movement” (Fig. 4e). Overall, the annotations represent the trends of the specification of the tissues at distinct developmental stages.

### Selection of potential functionally relevant inner ear lncRNAs

After the annotation process, we established a set of criteria to determine the relevant lncRNAs for further study. This included levels of expression and proximity to genes associated with deafness. In particular, we examined the developmental stage-specific expression of lncRNAs at either E16.5 or P0, and expression in the auditory system, vestibule, and/or cochlea. We assessed the expression of the candidate genes in other mouse tissues available through the UCSC Genome Browser, to learn about their differential expression. We studied the genomic context of the transcripts and the identity of their flanking genes. It has been suggested that the transcription of mRNAs and lncRNAs is closely regulated, leading to a *cis*-regulatory relationship between the two transcripts^18–20^. Therefore, we wanted to determine whether any lncRNA genes might be regulating gene expression in *cis*. We thus considered lncRNAs that are found up to 4 Mb from a gene of interest. lncRNAs were found in proximity to numerous genes crucial for the development and maintenance of the inner ear and several of them are associated with deafness. We examined whether any lncRNA genes are expressed in proximity to such genes, suggesting they may be involved in the genes’ regulation. For this purpose, a list of genes associated with “impaired hearing” (MP:0006325) and “deafness” (MP:0001967) were downloaded from the Mouse Genome Informatics (MGI) database (Supplementary Table S7).

**Figure 5:**
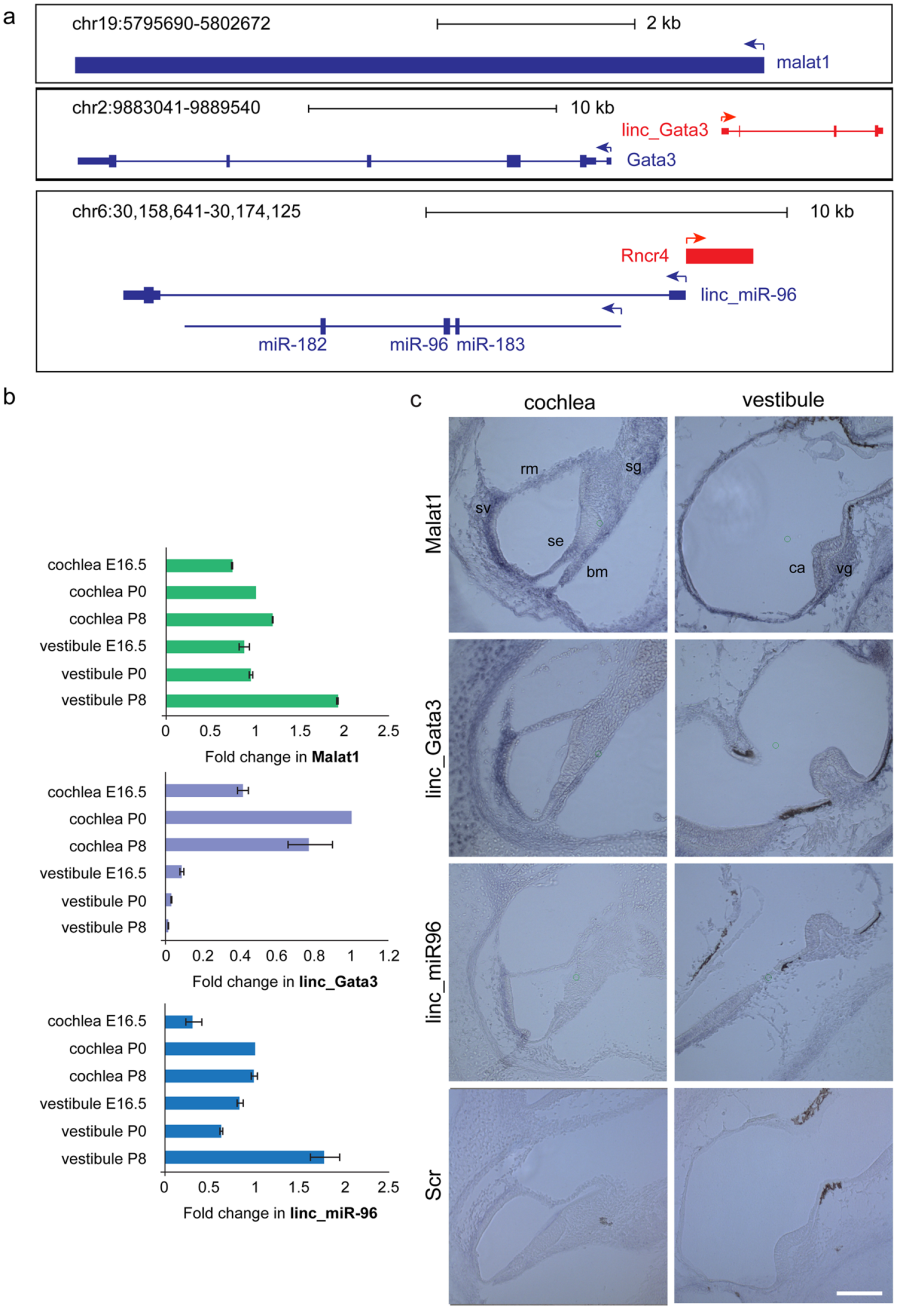
Spatial and temporal expression of lncRNA candidates in the inner ear. (**a**) Graphical representation of the genomic locus of Malat1, linc_Gata3, and linc_miR96. (**b**) Expression of three lncRNA candidates Malat1, linc_Gata3, and linc_miR96 in the developing inner ear, probed at three developmental stages (E16.5, P0, and P8) using qRT-PCR. (**c**) Specific expression patterns for lncRNAs by ISH at P0. Whole-mount inner ears were hybridized with LNA probes, followed by cryosectioning. For each lncRNA, images of the cochlear organ of Corti and vestibular crista ampullaris are shown. The bottom panel shows the scrambled (Scr) control LNA probe. Vestibular dark cells are present in all images of the vestibule, including the negative control. Scale bars: 100 μm. Abbreviations: se, sensory epithelium; rm, Reissner’s membrane; bm, basilar membrane; sv, stria vascularis; sg, spiral ganglion; ca, crista ampullaris; vg, vestibular ganglion.

In general, thirteen lncRNAs fit one or more of the above criteria (Table 1). Several lincRNAs were found in proximity to genes that are essential for inner ear development and maintenance, as well as are involved in disease. Since these lincRNAs are novel, we named them according to the gene to which they are adjacent. These include linc_Gata3, linc_Sox9, linc_Myo6, and linc_miR96 (Table 2; Fig. 5a). lncRNAs that were not previously described are named Ear-lincs. As expected, all the deafness genes were also expressed in our dataset (mean FPKM across all samples >15 for all cases, Supplementary Table S8). In 11/25 cases we observed a positive correlation between the lncRNA and the coding gene expression pattern across our 12 samples (R > 0.3, P < 0.05 in 8 of those, Supplementary Table S8). Interestingly, in three of the cases we observed a strong negative correlation (R < −0.3, P < 0.05 for 2 cases; Supplementary Table S8).

**Table 1:** Novel and known lncRNAs expressed in the inner ear.

	lncRNA	ID	Alias	Chromosomal position
**High in both**				
1	linc_Sox9	XLOC_006823	BC006965	chr11:112,663,919-112,781,976
2	linc_Tle1	XLOC_028537	AK042990	chr4:72,201,348-72,215,352
**Differentiating**				
**High in cochlea**				
3	linc_Myo6	XLOC_044667	D430036J16Rik	chr9:81,631,551-81,644,629
4	Ear-linc5	XLOC_039453		chr7:132,405,981-132,426,153
5	linc_Gata3	XLOC_023194	9230102O04Rik	chr2:9,883,041-9,889,540
**High in vestibule**				
6	linc_Mitf	XLOC_036924		chr6:98,053,893-98,064,929
7	linc_Gfi1	XLOC_032044	AK146255	chr5:107,725,157-107,727,104
8	Ear-linc2	XLOC_008975		chr12:79,270,851-79,275,604
9	Ear-linc1	XLOC_014079		chr14:100,974,673-100,981,692
10	Ear-linc3	XLOC_002473		chr1:80,439,379-80,461,513
11	Ear-linc4	XLOC_014052		chr14:98,746,148-98,809,358
High at E16.5				
12	Ear-linc8	XLOC_029914		chr4:54,013,948-54,032,486
**High at P0**				
13	Pantr1	XLOC_002128		chr1:42,648,200-42,694,825

**Table 2:** lncRNA candidate genes that are found in proximity to MGI hearing and deafness genes.

Gene ID	Alias	Chromosomal position	Deafness gene	Deafness gene name	Distance of lncRNA from deafness gene
XLOC_008505	2810410L24Rik	chr11:120,187,951-120,189,982	Actg1	Actin Gamma 1	0.15 Mb
XLOC_037026		chr6:114,125,712-114,131,136	Atp2b2	ATPase, Ca^++^ Transporting, Plasma Membrane 2	2 Mb
XLOC_012500		chr14:46,387,519-46,389,282	Bmp4	Bone Morphogenetic Protein 4	Within
XLOC_043384	Gm16675	chr8:46,728,377-46,739,515	Casp3	Caspase 3, Apoptosis-Related Cysteine Peptidase	88 Kb
XLOC_035799	Lockd	chr6:134,929,092-134,956,798	Cdkn1b	Cyclin-Dependent Kinase Inhibitor 1B	3.5 Kb
XLOC_023731	Dlx1as	chr2:71,530,638-71,537,891	Dlx1	Distal-Less Homeobox 1	Antisense
XLOC_021121	Emx2os	chr19:59,425,104-59,458,635	Emx2	Empty Spiracles Homeobox 2	Overlapping divergent
XLOC_023194		chr2:9,883,041-9,889,540	Gata3	GATA Binding Protein 3	4.4 Kb
XLOC_032044		chr5:107,725,157-107,727,104	Gfi1	Growth Factor Independent 1 Transcription Repressor	Overlapping divergent
XLOC_012867		chr14:57,109,201-57,112,912	Gjb2	Gap Junction Protein, Beta 2	249 bp
XLOC_005093		chr10:87,626,925-87,708,272	Igf1	Insulin-Like Growth Factor 1	0.15 Mb
XLOC_036116		chr6:30,158,641-30,174,125	miR-96	MicroRNA 96	Within the intron
XLOC_044667		chr9:81,631,551-81,644,629	Myo6	Myosin VI	1.3 Mb
XLOC_019183		chr18:42,398,395-42,461,349	Pou4f3	POU Class 4 Homeobox 3	2 Kb
XLOC_030934		chr4:150,565,574-150,568,859	Rere	Arginine-Glutamic Acid Dipeptide (RE) Repeats	Antisense
XLOC_042751		chr8:89,042,908-89,071,547	Sall1	Spalt-Like Transcription Factor 1	Overlapping divergent
XLOC_022976	AK052878	chr2:168,766,143-168,768,108	Sall4	Spalt-Like Transcription Factor 4	Overlapping divergent
XLOC_008931		chr12:73,049,034-73,061,044	Six1	SIX Homeobox 1	2.3 Mb
XLOC_011851		chr13:74,008,030-74,010,265	Slc12a7	Solute Carrier Family 12 (Potassium/Chloride Transporter), Member 7	0.2 Mb
XLOC_016930		chr16:90,188,039-90,203,810	Sod1	Superoxide Dismutase 1, Soluble	17 Mb
XLOC_025249	Sox2ot	chr3:34,638,252-34,680,851	Sox2	SRY (Sex Determining Region Y)-Box 2	Overlapping
XLOC_026712		chr3:34,663,511-34,665,217	Sox2	SRY (Sex Determining Region Y)-Box 2	11 Kb
XLOC_017828		chr17:70,834,664-70,836,044	Tgif1	TGFB-Induced Factor Homeobox 1	8 Kb
XLOC_020834	AK137243	chr19:21,161,683-21,172,091	Tmc1	Transmembrane Channel-Like 1	0.16 Mb
XLOC_015305	AK131739	chr15:78,911,966-78,913,660	Triobp	TRIO And F-Actin Binding Protein	34 Kb

Moreover, we examined which lncRNAs might be candidates for genes associated with deafness. The regions were derived from the list of loci with unknown genes in the Hereditary Hearing Loss Homepage (http://hereditaryhearingloss.org/). Chromosomal regions, based on microsatellite markers or coordinates of cytogenetic bands, were found in the relevant manuscripts, identified in hg19, and then converted to the homologous mouse interval (mm10). We found 2,019 lncRNA transcripts from 784 distinct genes mapped to regions associated with deafness in human where the causal gene is not presently known (Supplementary Table S9).

### Temporal and spatial expression of lncRNAs in the inner ear

To learn more about the candidate lncRNAs, we chose to focus on a subset of genes and study their spatio-temporal expression in mouse tissues using qRT-PCR. The expression of Malat1 was relatively high in all other mouse tissues studied (Supplementary Fig. S2a), similar to previous results^21, 22^. The expression of lincRNA_Gata3 was extremely high in the spinal cord, compared with other tissues (Supplementary Fig. S2b). The levels of linc_miR96 were considerably high in the mouse eye, and moderately expressed in lung, salivary gland, and spinal cord (Supplementary Fig. S2c). Retinal noncoding RNA 4 (Rncr4), a lncRNA contained within the linc-miR96 locus, was highly expressed in the eye, with more moderate expression in the lung, salivary gland, and spinal cord as well (Supplementary Fig. S2d).

Next, we examined the spatio-temporal expression in mouse cochlear and vestibular sensory epithelium. The expression of linc_Gata3, linc_miR96, and Malat1, detected by RNA-seq, was validated by qRT-PCR analysis (Fig. 5b). We found these lncRNAs to be expressed in the mouse auditory and vestibular sensory epithelia at E16, P0, and P8. The expression of Malat1 was dynamic and increased with age, exhibiting higher expression at P8 compared with P0 and E16.5, and at P0 compared with E16.5 in both auditory tissues examined (P < 0.005). The expression of linc_Gata3 was dynamic as well and was considerably higher in the cochlea compared with the vestibule. The expression peaked in the cochlea at P0 and gradually declined in the vestibule throughout development (P < 0.005). The expression of linc_miR96 was also dynamic and followed inverse patterns of expression in the vestibule and the cochlea; whereas the expression in the cochlea peaked at birth, the expression of linc_miR96 was the highest at P8 in the vestibule.

The spatial patterns of expression of Malat1, linc_Gata3 and linc_miR96 were evaluated in the auditory and vestibular sensory epithelia at P0 using *in situ* hybridization (ISH; Fig. 5c). We found all three lncRNAs to be expressed in cells of the auditory and vestibular systems. Specifically, in the cochlea the expression was evident in the sensory epithelium, as well as in the basilar membrane, in cells of the spiral prominence, in Reissner’s membrane, and in the apical surface of the spiral limbus. All lncRNAs were appreciably more highly expressed in the stria vascularis (Fig. 5c). In the vestibule the expression of the lncRNAs was found in all cells of the neuroepithelium. In addition, the expression of all candidates was more prominent in the neurons of the vestibular and spiral ganglia. Collectively, these results reveal that the inner ear lncRNAs are expressed in a temporal and spatial manner.

## Discussion

With the advancement of NGS, novel deafness genes and pathogenic variants in known genes are being discovered with relative ease^23, 24^. However, there are still a substantial number of unsolved familial deafness cases, even with the use of whole exome and/or whole genome sequencing. This led us to study the non-coding component of the transcriptome, which accounts for >98% of the genome, in an effort to find novel genomic regulatory elements^25^. Identifying such players can ultimately aid in isolating pathogenic variants or regulatory elements that can serve as the basis of human hearing and balance disorders.

In previous years, transcriptomic studies focused on analyzing the expression of coding gene transcripts. The mRNA levels of these transcripts were used to study expression patterns that provided clues about the functions of the translated proteins^26^. The rapid evolution of NGS technologies enabled an unparalleled advance in transcriptomic research. This included the discovery and characterization of various new classes of RNA molecules, among them microRNAs, endogenous siRNAs and circRNAs^27–29^. Another prominent class of RNA molecules discovered was the lncRNAs, found to play a critical role in cellular processes such as, but not limited to, differentiation, development and apoptosis^1, 30–32^. lncRNAs can act in genomic imprinting (for example, Xist, for silencing processing of the mammalian X-chromosome^33^) or as activators of gene expression (for example, Evf2 in activating Dlx2^34^). They can function in *cis* (such as Xist in repressing Xi^33^) or in trans (for example, HOTAIR^4^), and carry out their function in the nucleus or cytoplasm.

The transcriptomic repertoire of the mouse inner ear was studied previously^35–37^. However, to the best of our knowledge, little is known about lncRNAs in this tissue, other than a few specific lncRNAs^38, 39^. In the first comprehensive analysis of lncRNA expression in the inner ear of mice, we identified 6,318 lncRNA transcripts (Fig. 3a). Of these, more than 20% (1,460 transcripts) were not previously annotated and were considered novel. In accordance with previous studies, a comparative analysis of our newly identified lncRNAs in the mouse inner ear revealed characteristics that are shared with those in other mammalian tissues. As such, they are expressed at lower levels, consist of fewer and shorter exons, and they are less conserved than are protein-coding transcripts (Fig. 3b–d, Supplementary Table S1). Based on these results, we hypothesize that many more specific lncRNAs remain to be identified in other tissues and cell types.

In this study, we compared two sensory epithelia in the inner ear: the cochlea and vestibule. They are similar with respect to morphological and mechanotransduction properties of the hair cells, but at the same time, they also differ from one another. Neonatal and adult vestibular epithelial cells possess limited regenerative capabilities, a property that the mammalian cochlear epithelium lacks entirely (reviewed in ref. 40). Moreover, on the apical side of hair cells in both organs there are stereocilia bundles composed of actin; however, these projections are arranged and shaped differently in these two tissues^41, 42^. Another distinguishing feature between the cochlea and vestibule lies in the timing of tissue maturation, both in terms of sensory cell number and gain of mechanosensitivity. Neurosensory hair cells found in the mouse auditory sensory epithelium are formed between E13.5 and P0 and consequently acquire sensory transduction by P2^43, 44^. In contrast, vestibular hair and supporting cells exit the cell-cycle and differentiate between E15 and P14^41^. These hair cells become mechanically sensitive much earlier, from E17^45^. In both systems, the processes of terminal differentiation and cell-cycle exit occur in a distinct spatial pattern, apex-to-base and central-to-peripheral gradients in cochlea and vestibule, respectively, which in the cochlea leads to a two-day delay in the process between base versus apex^8, 46^.

Importantly, we observed that a clear bias exists regarding the number of significant DEGs between the cochlea and vestibule, with the cochlea having more DEGs (Fig. 4a,b). We speculate that this can be attributed to the fact that each tissue has a very distinct “window” for acquiring mechanosensory properties. In the vestibule this window is somewhat wide; therefore, it may lead to a continuous genetic program, whereby the genes are expressed over time. In contrast, the window in the auditory sensory organ is relatively short, suggesting that the genes responsible for the gain of mechanosensitivity should peak at their expression levels, especially in the E16.5 transcriptome. These differences between the neurosensory organs suggest the role of the differentially expressed lncRNAs and mRNAs between the cochlea and vestibule and can contribute to revealing the identities and functions of each of them.

To identify the major genetic events and processes occurring during the late embryonic development of the auditory and vestibular sensory organs, we examined the enriched GO annotations. This analysis showed that the enriched terms from E16.5 to P0 are highly associated with transcription and cell cycle processes (Fig. 4c). More specifically, at E16.5, both the cochlea and the vestibule undergo major specification events and the number of cells in the epithelium expands. Therefore, it is not surprising that the enriched GO annotations at P0, compared with E16.5, can be distinguished between the two sensory organs (Supplementary Table S6).

In addition, we observed that there are GO annotations that are enriched in the cochlea compared with the vestibule and vice versa, at both E16.5 and P0. We posit that the reason for the up-regulation of genes associated with neuronal processes in the cochlea at all developmental stages probed (Fig. 4e) is once more linked to the window of maturation that the tissue undergoes during this period. Interestingly, the potential contribution of the immune response throughout normal development of wild-type vestibular tissue is evident from the enrichment of transcripts with annotations related to the immune system (Fig. 4d). Although some immune and inflammation regulatory pathways have been described previously in the inner ear, their role in the sensory vestibular organs has not been addressed extensively and therefore is largely unknown.

A number of lncRNAs were examined further, due to their proximity to deafness genes, including linc_Gata3 and linc_miR96. Gata3, a zinc-finger transcription factor is believed to have a role in cochlear wiring, and more specifically, guiding spiral ganglion neurons^47^. *GATA3* pathogenic variants are associated with deafness, most commonly in the form of a HDR syndrome (for hypoparathyroidism, sensorineural deafness, renal anomaly)^48^. The majority of reported cases have at least two of the three phenotypes, although there is at least one documented case of a *GATA3* variant associated only with sensorineural hearing loss^49^. Mutations in the seed region of miR-96 have been found to cause progressive non-syndromic hearing loss in mammals^50, 51^. However, the underlying molecular mechanism that leads to this pathology has not been fully elucidated. linc_miR96 contains the pri-microRNA for the mir-183/96/182 cluster in its intron (Fig. 5a). In addition, apart from being expressed in the inner ear, based on qRT-PCR and UCSC analyses, it is expressed in the central nervous system (CNS) at E11.5 and E14, as well as in the bladder, eye, and placenta. In the same locus, an additional lncRNA, retinal noncoding RNA 4 (Rncr4), was recently recognized to be expressed in maturing photoreceptors, where it is divergently expressed, compared with the polycistronic miR-183 family^52^. This lncRNA was identified as a factor that stimulates pri-miR-183/96/182 processing. Rncr4 is also expressed in our RNA-seq data (Supplementary Fig. S2d) and miR-182 and miR-183 are notably highly expressed in both the vestibule and cochlea at both developmental stages^36^. Consequently, we hypothesize that the two transcripts, linc_miR96 and Rncr4, might play a role in the transcriptional regulatory program in the miR-96 locus. As a result, studying these lncRNAs in the inner ear might help elucidate the mechanisms of action in this important locus.

There are severe limitations in studying ncRNAs in human tissue, since it is mostly inaccessible and there are no reliable and sufficiently representative cell lines. Therefore, we have worked with mouse inner ear tissue, which is highly similar to the human organ, both developmentally and functionally^53^. Since our goal is to provide relevant medical information regarding human inner ear function and disorders, we ultimately want to study orthologous lncRNAs expressed in humans. A recent report identified ncRNAs from the inner ear tissue of three adult individuals with tumors^15^. Because lncRNA expression is highly regulated during development, it is perhaps no surprise that our comparison of mouse embryonic and human adult lncRNAs orthologues failed to uncover many conserved transcripts. Still, we found 93 pairs of orthologues, with 22 of them clustering together and exhibiting high expression profiles, either in human or cross-species. We hypothesize that these lncRNAs are likely to have a functional role in the inner ear.

In addition, it will be relevant to study lncRNA expression at a higher resolution of the tissue. Single-cell approaches have already been used in the inner ear^35, 54^. In these attempts, it was possible to study two aspects in the complex architecture of the cochlea: examining genes that are expressed in gradients along the apex-to-base axis or between different cell subpopulations. To date, use of single-cell technologies in the inner ear has been coupled with qRT-PCR, where a pre-selected set of genes is studied. However, rapidly evolving advancements in transcriptomic technologies, including ultra-sensitive and low-input methodologies and improved bioinformatic methods, have already employed both RNA-seq on single cells (scRNA-Seq)^55^ as well as *in-situ* RNA-seq^56–59^. These technologies could be used in the future to study lncRNAs and their role in the gradient-like expression patterns in the cochlea as well as in the vestibule.

Further analysis of mouse auditory lncRNAs is essential. The poly-A tail transcript selection approach used here restricted our ability to discover a more in-depth lncRNA profile. The expression levels of lncRNAs are at least one order of magnitude lower than those of coding genes (Fig. 3d)^60, 61^. Therefore, the depth of RNA sequencing used may be insufficient for capturing lncRNAs with lower levels of expression. In addition, to decipher the functional roles of the discovered lncRNAs in the context of the whole tissue, a deeper analysis is required for identifying both their subcellular localization and cell-specific expression pattern^62^. The lncRNA transcripts with cell-specific expression patterns may have explicit functional roles in diverse cellular processes within the inner ear.

There are still numerous open questions regarding the inner ear’s morphology and physiological features. Where might lncRNAs be involved in regulatory processes in the inner ear during the period we examined? We identified lncRNAs in sensory epithelia derived from cochleas and vestibules at E16.5 and at birth. We have chosen these time points as they represent stages of morphological and cell specification events in the developing mammalian inner ear. Key regulatory points may be associated with cellular processes such as plasticity, regeneration and apoptosis, which require strict temporal and spatial regulation. Unraveling the molecular mechanisms governing lncRNAs of the transcriptome may contribute to deciphering the genetic basis of deafness in unsolved families. Moreover, these lncRNAs may also serve as a rich source for antisense oligonucleotide therapeutics of non-coding target genes.

Finally, our study provides the first lncRNA catalogue, which is highly beneficial for elucidating the developmental programs occurring in the mouse inner ear. We anticipate that this catalogue will serve as a valuable reference for future research into the involvement of lncRNAs in neurosensory systems.

## Methods

Additional methods are available on-line in the Supplementary Materials section.

### Animals

C57BL/6J mice, including newborns, adults, and time-mated pregnant females, were purchased from Envigo, Jerusalem, Israel. All procedures involving animals met the guidelines described in the National Institutes of Health Guide for the Care and Use of Laboratory Animals and have been approved by the Animal Care and Use Committee of Tel Aviv University (M-13-114).

### RNA sequencing

Sensory epithelia were isolated from the cochlea and vestibule of E16.5 and P0 C57BL/6 mice. For each age, RNA was pooled from 10 mice. RNA was extracted using the RNeasy Micro Kit (Qiagen). The integrity of the total RNA was estimated using the 2100 Bioanalyzer (Agilent Technologies). Next, 400 ng of total RNA was used to prepare twelve paired-end (PE) cDNA libraries using the TruSeq Stranded mRNA Sample Prep Kit (Illumina) at the Tel Aviv University Genome Analysis Laboratory (https://en-med.tau.ac.il/Genomic-Analysis-Lab). The libraries were sequenced to obtain PE strand-specific 100 bp reads on a HiSeq. 2500 (Illumina) at the Technion Genome Center, Haifa, Israel.

### lncRNA identification and differential expression analysis

Reads were aligned to the mouse genome (10 mm assembly) using STAR based on splice junctions from the Ensembl database^11^. Transcriptome was assembled using CuffLinks^12^ and further processed using the PLAR method^10^. Transcript abundance was estimated using RSEM^14^ and differentially expressed genes were identified using DeSeq. 2^16^. GO analysis was performed using the Bioconductor topGO package^17^. Conserved lncRNAs were identified as described using the UCSC browser mm10 assembly whole genome alignment and direct comparisons using BLASTN^63^.

### *In-situ* hybridization

At least three independent *in-situ* hybridization (ISH) experiments were performed with each probe, and at least three inner ears were included in each experiment. We performed whole-mount ISH of the inner ears as previously described. Briefly, newborn C57BL/6 mouse inner ears were dissected and fixed in 4% paraformaldehyde (PFA). Hybridization was carried out overnight with 25 nM custom-designed 6-FAM (fluorescein)-labeled LNA^TM^ probes (Exiqon), at 20–22 °C below the melting temperature of the probe. LNA probe sequences are available upon request. The LNA^TM^ probes were detected by anti-FAM-AP (alkaline phosphatase conjugated) antibody (Roche). NTB/BCIP (Sigma) was used for the colorimetric detection of AP. Hybridization was also performed with an LNA-scrambled probe, as a negative control. Next, the tissues were cryosectioned into 10 μm sections using a LEICA CM3050S cryostat. Finally, the sections were mounted and images were taken using an ArcturusXT™ Laser Capture Microdissection (LCM) instrument.

### RNA isolation and qRT-PCR

Inner ears or the sensory epithelium of the cochlea and vestibule were dissected, frozen in liquid nitrogen, and stored at −80 °C. Total RNA was extracted by the RNeasy Plus Mini Kit (Qiagen) and diluted in RNase-free water (Life Technologies). The Mouse Total RNA Tissue Panel (Clontech) was used to examine the presence and the level of the expression of lncRNAs in various tissues. cDNA was prepared from 1 ug of RNA using the High Capacity cDNA Reverse Transcription Kit with random hexamers (Applied Biosystems). mRNA expression was evaluated using the Fast SYBR® Green Master Mix (Applied Biosystems) in the StepOneTM Real-Time PCR System (Applied Biosystems).

Primers were designed for 80–150 base-pair (bp) segments using Primer3 (http://bioinfo.ut.ee/primer3/). All primers were validated, including the amplification efficiency and correlation coefficient (R^2^) of each primer pair; samples were examined using a standard curve with five cDNA dilutions. In addition, a melt curve was performed to verify the specificity of the primers. Primers are available upon request. No template control (NTC) samples were included as negative controls. The 2^−ΔΔCt^ method was used to calculate the expression of each lncRNA. mRNA expression was normalized to Gapdh. Cochlear tissue from postnatal day 0 was used as the control sample. The data in the figures are presented as the mean ± SD.

### Data availability

The four RNA-seq datasets were deposited in NCBI and are available in the Gene Expression Omnibus (GEO) repository under accession no. GSE97270 (E16.5) and no. GSE76149 (P0) and are available on the gene Expression Analysis Resource web portal, gEAR, http://umgear.org/p?s=ace02363 (SVG); http://umgear.org/p?s=1e3f9408 (bar graph).

## Electronic supplementary material

Supplementary Information

Supplementary Table S1

Supplementary Table S2

Supplementary Table S3

Supplementary Table S4

Supplementary Table S5

Supplementary Table S6

Supplementary Table S7

Supplementary Table S8

Supplementary Table S9
