# Supplementary material for: Genome-wide identification and expression profiling of long non-coding RNAs in auditory and vestibular systems
Source: Sci Rep. 2017 Aug 17;7:8637. doi: 10.1038/s41598-017-08320-3 (PMC5561060; doi:10.1038/s41598-017-08320-3)
Supplement: Supplementary file 1 — Supplementary Information [file 41598_2017_8320_MOESM1_ESM.pdf]

Genome-wide identification and expression profiling of long non-coding RNAs in auditory and vestibular systems

Kathy Ushakov, Tal Koffler-Brill, Aviv Rom, Kobi Perl, Igor Ulitsky, & Karen B Avraham

Supplementary Information

CONTENTS

Supplementary methods……………………………………………………………..………..…2
Titles of supplementary tables…………………………………………………………………..3
Supplementary figures………………………………………………………….………………..4

Supplementary methods

Comparison of mouse and human lncRNAs. The mouse and human datasets contained 3,239 and 7,109 lncRNAs, respectively, measured across different conditions. In both datasets, the unit of measurement was FPKM (Fragments Per Kilobase of transcript per Million). For each dataset, we performed a $\log_2$ transformation on the data, centered the measurements on 0, and used median to aggregate measurements between replicates. This normalization procedure was performed in order to allow comparison of measurements between species. Then, we matched human lncRNAs to their orthologous mouse genes. Orthology between lncRNAs was based on both sequence similarity and synteny. A pair of human and mouse lincRNAs were classified as orthologs if their BLAST aligning score (E) was under 10^{-5}, and both appeared in the same synteny block. The parameters for BLAST and synteny blocks reconstruction were selected based on calibration experiments[1]. The reference human and mouse genomes used were hg19 and mm10, respectively. We resolved multiple matching of mouse lncRNAs to a single human lncRNA by selecting the match for which we had less missing measurements, resolving ties by preferring a higher average expression. The lncRNAs were then clustered into groups according to their expression patterns across species, tissues and ages. The choices of clustering method and optimal number of clusters were chosen to maximize the average silhouette using the dendextend package (http://cran.r-project.org/web/packages/dendextend/index.html). Tested methods included `"ward.D"`,`"ward.D2"`, `"single"`, `"complete"`, `"average"`(= UPGMA), `"mcquitty"` (= WPGMA), `"median"` (= WPGMC) or `"centroid"` (= UPGMC). The maximal number of clusters allowed was ten. The chosen method was `"ward.D"` and number of clusters was three. The heatmap was generated in R Bioconductor using the heatmap.2 function of the gplots package (http://cran.r-project.org/web/packages/gplots/index.html).

1. Hezroni, H. *et al.* Principles of long noncoding RNA evolution derived from direct comparison of transcriptomes in 17 species. *Cell Rep.* **11,** 1110–22 (2015).

Titles of supplementary tables

Table S1. RNA-seq reads to identify lncRNAs and coding genes from cochlear and vestibular sensory epithelium.

Table S2. Exon coordinates of lncRNA transcripts derived from cochlear and vestibular sensory epithelium and novel lncRNA coordinates (BED format).

Table S3. Comparison of lncRNAs in human and mouse inner ears.

Table S4. Differentially expressed mRNA genes between different samples.

Table S5. Differentially expressed lncRNA genes between different samples.

Table S6. Enriched terms following gene ontology (GO) enrichment analysis of differentially expressed genes between different samples.

Table S7. List of genes associated with mammalian phenotypes (MP) "impaired hearing" and "deafness", as downloaded from Mouse Genome Informatics (MGI).

Table S8. Expression correlation between lncRNAs and their adjacent deafness-associated genes.

Table S9. Mouse lncRNAs mapped to syntenic chromosomal locations of human deafness loci.

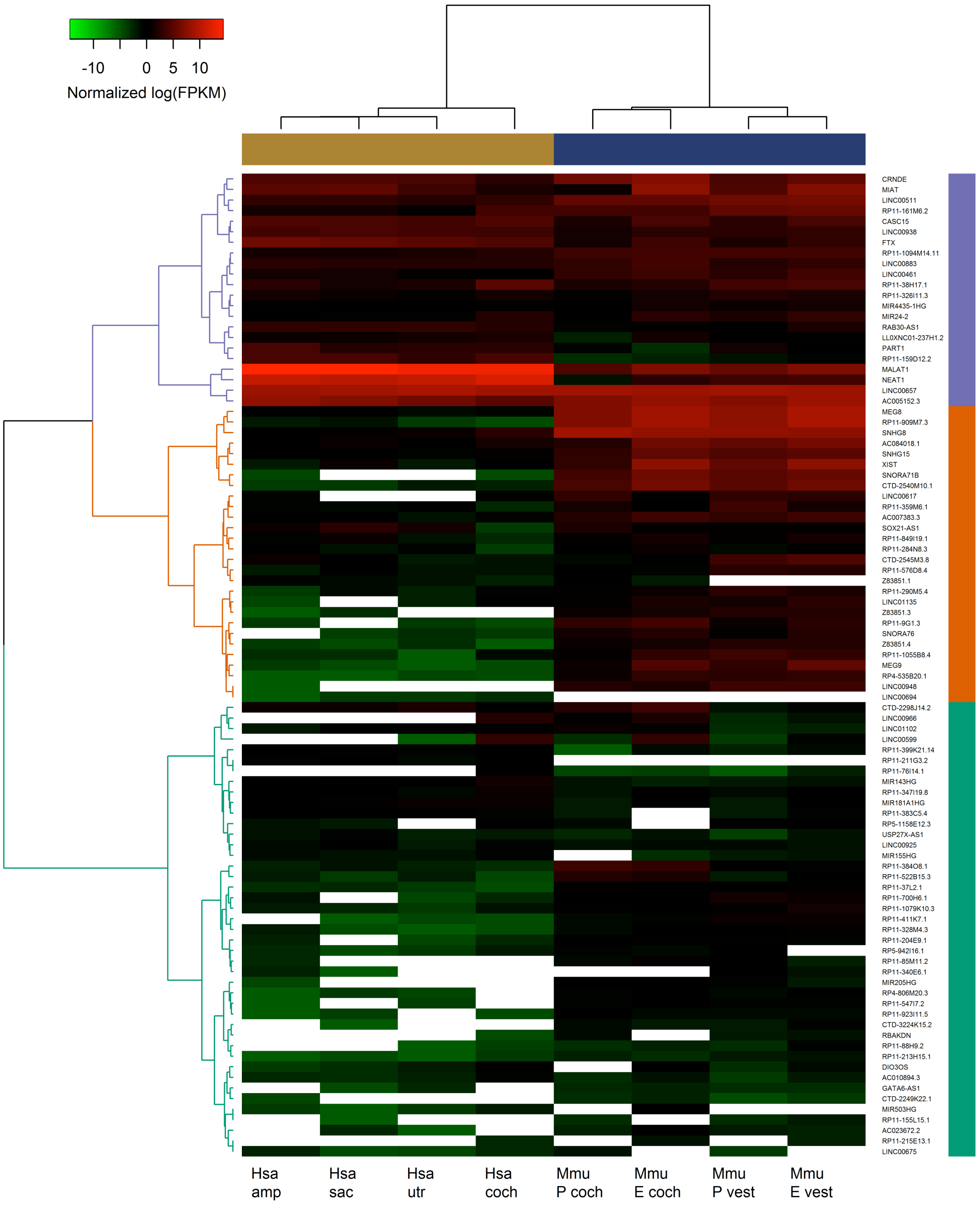

Figure S1. Cluster heatmap of inner ear human-mouse lncRNA orthologs. Comparison of orthologous human and mouse lncRNAs according to their spatial and temporal expression. For 93 human lncRNAs expressed in the inner ear with syntenic homologs in mouse, their normalized expression is shown across species, tissues and ages. Human and mouse samples are marked on the horizontal side bar in gold and blue, respectively. lncRNAs were clustered into three groups based on their expression pattern, with the assignment marked in purple, orange and green on the vertical branches and side bar. White tiles represent missing measurements. amp, ampula; sac, saccule; utr, utricle; coch, cochlea, vest, vestibule; P, postnatal; E, embryonic.

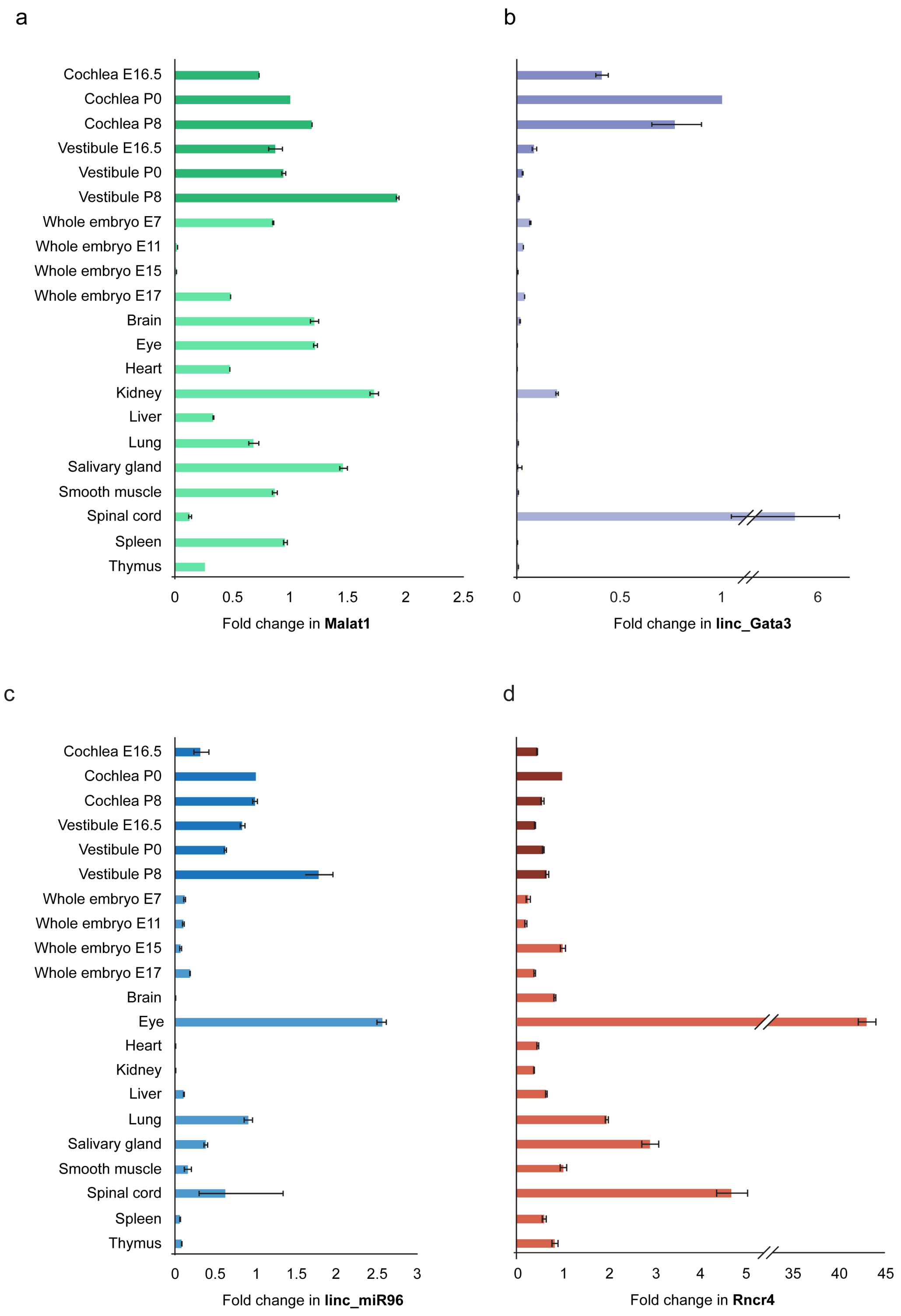

Figure S2. Temporal expression of lincRNA candidates in mouse tissues. Expression of three lincRNA candidates, namely, (**a**) Malat1, (**b**) linc_Gata3, (**c**) linc_miR96, and (**d**) Rncr4, in the developing inner ear, examined at three developmental stages (E16.5, P0, and P8; darker shades) and mouse tissue panel using qRT-PCR.
